# Post-discharge critical COVID-19 lung function related to severity of radiologic lung involvement at admission

**DOI:** 10.1186/s12931-021-01625-y

**Published:** 2021-01-21

**Authors:** Laurent Truffaut, Lucas Demey, Anne Violette Bruyneel, Alain Roman, Stephane Alard, Nathalie De Vos, Marie Bruyneel

**Affiliations:** 1 Department of Pneumology, CHU Saint-Pierre, Brussels, Belgium; 2 KiCarre, Research Department, Lentilly, France; 3 Department of Intensive Care Medicine, CHU Saint-Pierre, Brussels, Belgium; 4 Department of Radiology, CHU Saint-Pierre, Brussels, Belgium; 5 Department of Clinical Chemistry, LHUB-ULB, Université Libre de Bruxelles, Brussels, Belgium; 6 Université Libre de Bruxelles, Brussels, Belgium

## Abstract

Lung function impairment persists in 55% of critical COVID-19 patients three months after ICU discharge. Patient lung function, exercise capacity, radiologic, and quality of life data suggest impairment is related to radiologic lung involvement at admission.

## To the editor

Acute respiratory distress syndrome (ARDS) is observed in 5% of COVID-19 patients and requires intensive care unit (ICU) admission. Regardless of the aetiology, ARDS survivors remain disabled and have increased rates of specialist use and re-admission to the hospital and ICU, as well as higher healthcare costs [1]. Cognitive impairment and quality of life (QoL) also remain severely impaired in long-term ARDS survivors [2]. Abnormal chest radiography and pulmonary function results persist years after discharge but tend to improve at 5 years [3].

As critical mid- and long-term sequelae associated with COVID-19 are not yet known, we aimed to assess lung function (Pulmonary function test (PFT) and respiratory muscle strength measurements), exercise capacity (6-min walking distance test (6MWDT)), dyspnoea (modified Medical Research Council (mMRC) dyspnoea scale), and QoL (Short Form 36 (SF-36)) in ARDS COVID-19 survivors, 3 months after ICU discharge. A non-contrast enhanced chest CT was also performed, in order to assess radiological changes between hospital admission and post-discharge period in terms of number of affected segments in both lungs (maximal score = 20), abnormalities distribution (peripheral/ central location, diffuse) and predominant pattern (ground glass opacities (GGO), consolidation, fibrosis).

Between 3 March and 2 June 2020, 63 patients were admitted to our ICU with a COVID-19 diagnosis confirmed by PCR and/or chest CT Scan. A total of 23 died (37%), and 22/33 ARDS survivors were included in this study. All included patients provided written informed consent to participate in the study. The study protocol was approved by the Saint-Pierre University Hospital ethics committee (AK/16–01-18/4613).

The mean age was 54.6 ± 10.9 years, 64% required mechanical ventilation (MV), and ICU length of stay was 20.7 ± 16.7 days. Extracorporeal membrane oxygenation use was required in 2 patients. Symptoms duration before hospital and ICU admission was respectively 7.7 ± 1.4 and 10.4 ± 2 days.

Demographics and clinical data are summarized in Tables 1, 2.

**Table 1:** Demographics and clinical data of COVID-19 patients admitted to ICU

Age (mean ± SD)	54.6 ± 10.9
Sex Males	n = 16 (72.7%)
Obesity	
No Grade 1(BMI 30–34.9 kg/m^2^) Grade 2(BMI 35–39.9 kg/m^2^) Grade 3(BMI 40–49.9 kg/m^2^)	n = 11 (50%) n = 4 (18.2%) n = 6 (27.3%) n = 1 (4.6%)
Smoking history	
No Active Former	n = 18 (81.8%) n = 2 (9.1%) n = 2 (9.1%)
Alcohol abuse, %	0
Medical history	
Diabetes Hypertension HIV Cancer Obstructive sleep apnea -CPAP treated	n = 6 (27.3%) n = 10 (45.4%) n = 1 (4.6%) n = 1 (4.6%) n = 2 (9.1%) n = 2

CPAP: continuous positive airway pressure, BMI: body mass index

**Table 2:** Lung function, dyspnea, quality of life, and chest CT assessments in COVID-19 patients 3 months after ICU discharge

Pulmonary function test, n = 22 (mean ± SD)	FEV1 (L)	2.88 ± 0.66
	FEV1 (%)	89.4 ± 15.7
	FVC (L)	3.38 ± 0.81
	FVC (%)	83.64 ± 16.9
	FEV1/FCV	0.86 ± 0.06
	DLCO (%)	80.7 ± 14.3
PFT interpretation	Normal Restrictive pattern Restrictive + altered diffusion Altered diffusion	n = 10 n = 2 n = 4 n = 6
6MWDT, n = 20 (mean ± SD)	Distance (m)	514.4 ± 93.1
	Distance (%)	73.5 ± 12.3
	Distance < 80%	n = 13
	Desaturation	n = 6
Respiratory muscle strength, n = 21 (mean ± SD)	MIP (cm H_2_O)	85.7 ± 24.9
	MIP (%)	81.8 ± 15.2
	MIP < 80%	n = 9
	MEP (cm H_2_O)	102.8 ± 32.3
	MEP (%)	66.1 ± 30.7
	MEP < 80%	n = 17
MMRC, n = 21, mean ± SD		0.7 ± 1.7
SF-36, n = 22 (mean ± SD)	Physical functioning	70.79 ± 21.30
	Role physical	33.82 ± 37.44
	Pain	64.60 ± 26.61
	General health	59.90 ± 17.62
	Vitality	56.84 ± 16.68
	Social	69.38 ± 22.02
	Role emotional	38.61 ± 48.77
	Mental health	69.68 ± 16.99
Chest CT Scan, n = 22	*Baseline*	*3-months*
Normal	n = 0	n = 3
Number of affected segments in both lungs (mean ± SD), score/20	17.2 ± 19	8.1 ± 10.1
Predominant abnormalities		
Ground glass opacities	n = 22	n = 0
Consolidation	n = 16	n = 2
Fibrosis	n = 9	n = 19
Abnormalities distribution		
Peripheral location	n = 8	n = 7
Diffuse location	n = 14	n = 12
Lower lobes predominant involvement	n = 12	n = 11

SF-36: Short Form 36 questionnaire, mMRC: modified Medical Research Council dyspnea scale, ICU: intensive care unit, FEV1: forced expiratory volume in 1 s, FVC: forced vital capacity, DLCO: Diffusion capacity of the lung for carbon monoxide, MIP:maximum inspiratory pressure, MEP: maximum expiratory pressure

PFT remained abnormal in 55% of patients, exhibiting restrictive pattern ± altered diffusing capacity of the lungs for carbon monoxide (DLCO). Sixty-five percent exhibited a 6MWDT below 80% and 52% were free from exertional dyspnoea according to mMRC scale. QoL (SF-36) was worse in the domains of "limitations due to physical health problems" and "limitations due to emotional problems".

**Fig. 1:**
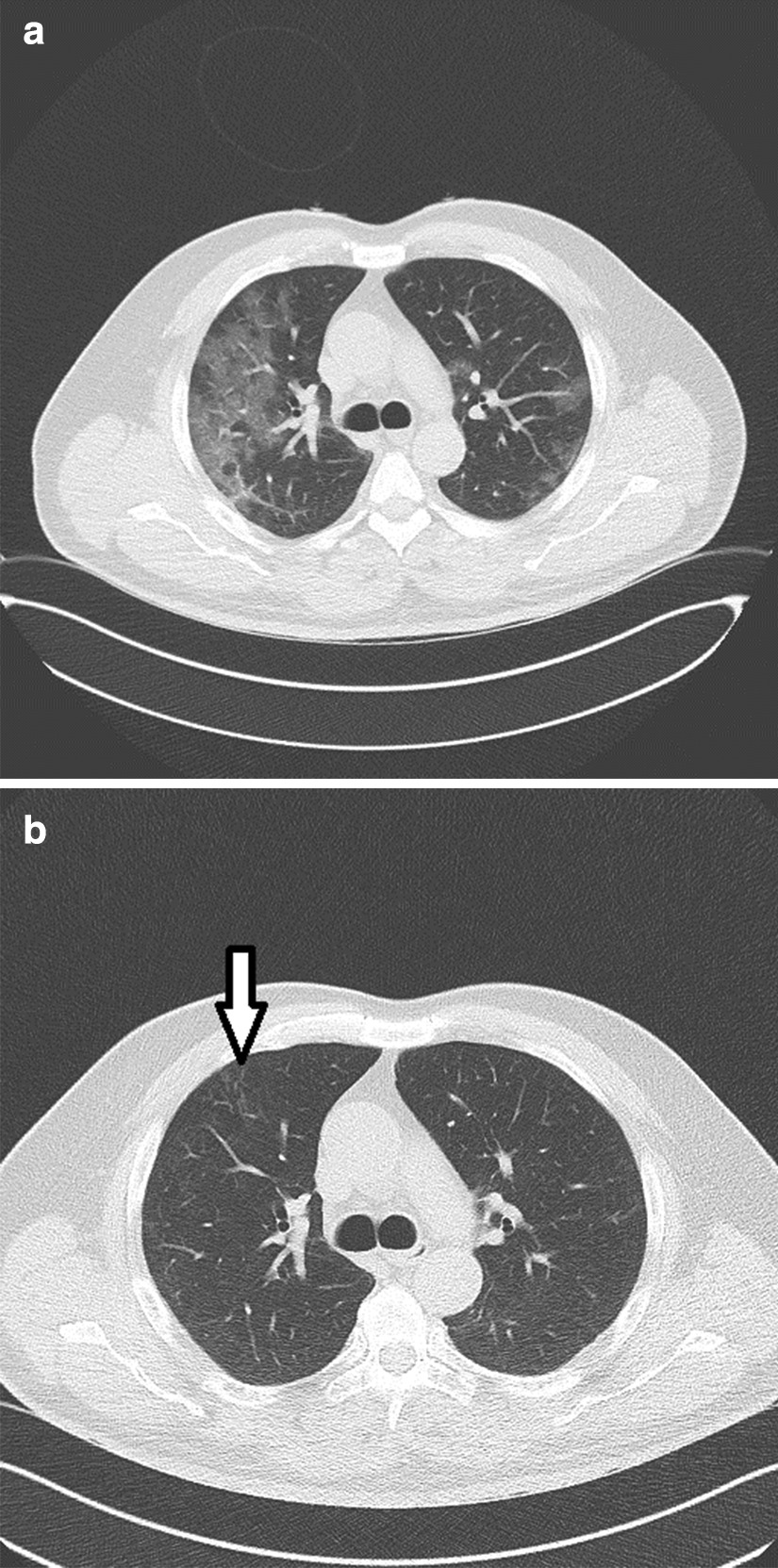
Chest CT Scans of a critical COVID-19 patient, at admission, 14 days after symptoms onset (**a**) and 3-months post ICU discharge (**b**). Bilateral ground glass opacities (GGO) in a peripheral distribution are observed in both upper lobes on Pannel A. On Pannel B, GGO has disappeared, replaced by a discrete fibrosis area in the upper right lobe (black arrow)

All but 2 patients exhibited a decrease in affected segments on chest CT and 3/22 (14%) showed normalization. The majority of abnormalities were ground glass opacities at hospital admission and fibrosis 3 months later. Results are reported in Table 1, Fig. 1.

On multivariate analysis, low total lung capacity was associated with the need for mechanical ventilation (p = 0.044) and low forced expiratory volume (FEV1) with high APACHE II score (p = 0.049). DLCO impairment, low FEV1, and number of affected segments on 3-month CT correlated with initial number of affected segments on chest CT scan (p < 0.0001, < 0.0001, and 0.01, respectively) but not with the initial radiologic pattern (GGO, consolidation, fibrosis). The use of glucocorticoids (GC) was associated with better outcomes in the above-mentioned parameters (p = 0.02, 0.008, and 0.04, respectively). In terms of demographic data, DLCO impairment and FEV1 were worse in males (p < 0.001 and 0.005).

**Fig. 2:**
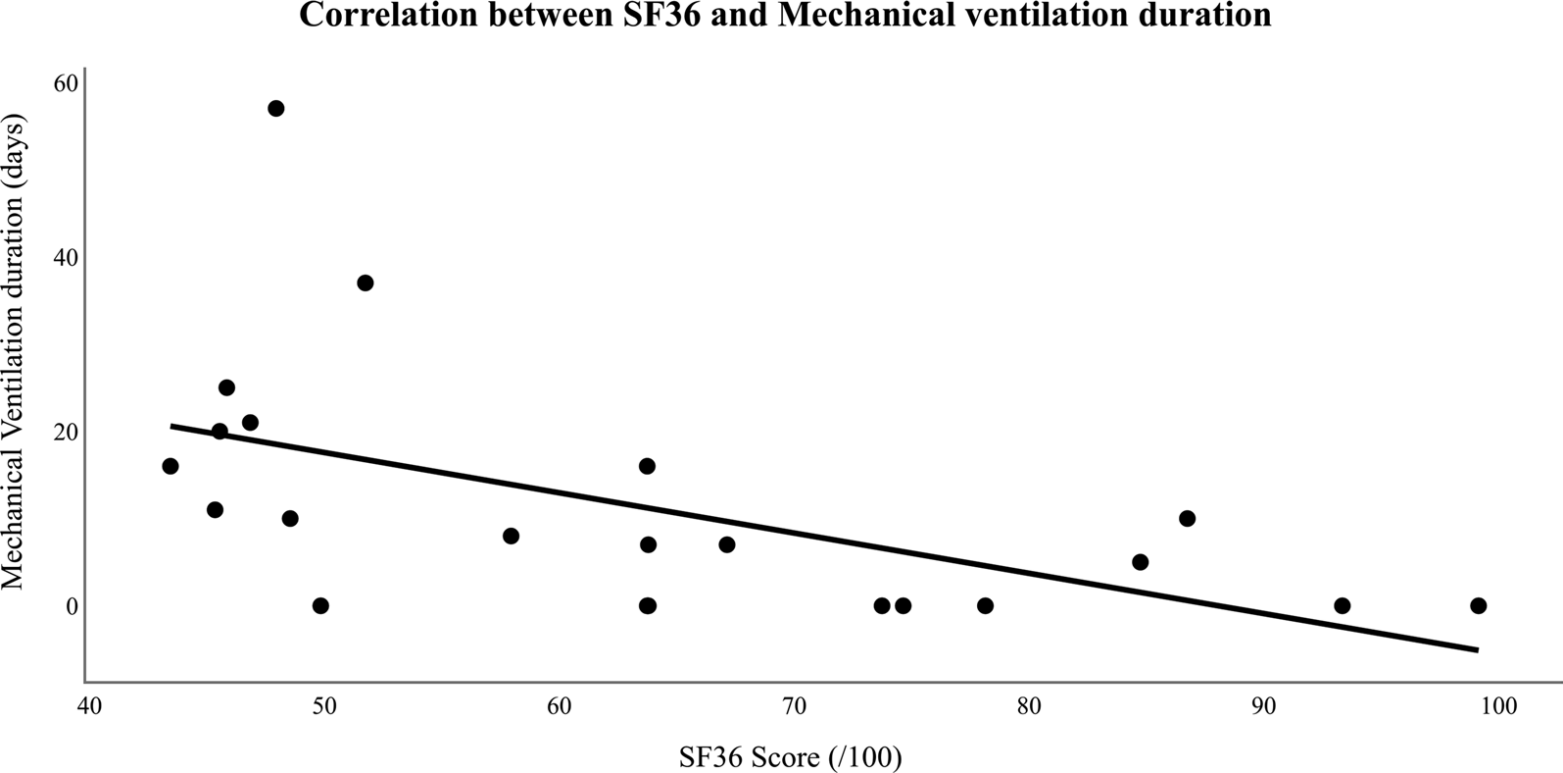
Correlation between SF-36 and mechanical ventilation duration

Regarding QoL, low SF-36 values correlated with APACHE II (p = 0.026), MV (p = 0.05), MV duration (p = 0.0004), and ICU length of stay (p = 0.0002) (Fig. 2). The only predictor of fibrosis on 3-months chest CT scan among demographic and medical data was the presence of obstructive sleep apnoea (p < 0.001).

This is the first report focusing on 3-months outcomes of critical COVID-19 survivors. Patients remained largely disabled 3 months after discharge in terms of lung function, exercise capacity, exertional dyspnoea, and QoL. Radiologic impairment was improved but normalization was only observed in a minority of patients.

Impaired DLCO has already been described as a common abnormality persisting after coronavirus infection. Indeed, SARS-CoV-2 looks like the two first coronavirus outbreaks in this regard (SARS CoV-1 in 2002 and MERS in 2012). In these patients, altered DLCO has been documented, with persisting significant impairment in DLCO in 24%-37% of survivors one year after illness onset [4]. More recently, in COVID-19 patients, DLCO impairment has been shown to be common and is observed in 39%-53% one month after discharge in hospitalized patients [5, 6] and in 16% 3 months after discharge [7]. We observed more decreased DLCO in our series, 45%, in line with the critical status of our patients. Indeed, in ARDS survivors, DLCO is the most often impaired lung functional marker, averaging 65% after 1 year [8]. A total of 27% of patients exhibited a restrictive pattern. This has been described in 12%-23% one month after discharge in COVID-19 hospitalized patients [5, 6].

As in other series, we observed a correlation between initial chest CT scan involvement and impaired PFT [5]. Previously, in SARS CoV-1, correlations between persistent chest X-ray abnormalities and impaired PFT were observed [9].

Regarding initial chest CT abnormalities, the different observed patterns can be explained by the delay between symptom onset and hospital admission. Indeed, GGO occurs generally since the beginning of COVID-19 and tends to decrease after 14 days. Consolidations appear around day 9 and fibrosis after day 14 [10, 11]. However, the severity of the disease seems rather related to the extent of lesions on initial chest CT than to the type of abnormalities [12, 13]. In the present study, we have shown that 3-months radiological and functional sequelae were related to initial disease extension on chest CT. Currently, there are no similar available reports.

With the generalisation of GC use, one can suppose that long-term recovery should be better, even in patients with severe radiological disease at admission. Indeed, in our series, better functional and radiologic outcomes were associated with GC use. At the time of hospitalization of our patients, GC use was controversial and not routinely advised. We have, however, administered GC to 7 patients in the context of persistent/refractory moderate-to-severe ARDS. Since this period, knowledge about COVID-19 has evolved with the publication of the RECOVERY study, highlighting mortality reductions associated with the use of GC in severe COVID-19 patients [14]. What we have observed in our series confirms the benefit of this treatment.

We have observed that obstructive sleep apnoea (SAOS) was a predictor of fibrosis on 3-months chest CT scan, although both patients were efficiently treated. It has been reported that SAOS is very prevalent in idiopathic lung fibrosis, and could induce fibrotic lesions through pleural pressure swings related to repeated upper airways collapse. These pressure swings lead to tractional microinjury of the alveoli, inducing further epithelial cells activation and fibroblast recruitment, favouring lung fibrosis [15].

Male sex was associated with worse PFT. This is not surprising as male sex has been established as a risk factor for developing ARDS, and thus a factor of disease severity, in a large Chinese series [16].

Exercise capacity, measured by 6MWDT, was still poor 3 months after ICU discharge and is probably multifactorial, according to the pre-existing medical conditions of the patients (obesity, diabetes,..), that are usually associated with decreased exercise capacity. We were not able to identify ICU parameters that predicted this impairment. In hospitalized SARS-CoV-1 patients, Ahmed et al. showed that 6MWDT was reduced at 3 months and slowly improved up to 12 months [17]. This disability was expected as critical illness can have important effects on the neuromuscular system. Acute lung injury survivors generally take 12 months to recover from physical complications [18]. However, maximum inspiratory pressure (MIP) was 82% 3 months after ICU discharge. This is much better than what was described in previous large series of patients experiencing prolonged MV, where MIP was 53% at discharge and increased to 68% 6 months later [19]. In a small, severe COVID-19 series assessed 30 days after discharge, the mean MIP was 80% [5], suggesting that recovery of respiratory muscle strength could be faster in COVID-19 than in other causes of acute respiratory failure. Of note, recovery was good in our patients despite the fact that half of them was suffering from obesity, which alters respiratory muscle strength.

Decreased QoL was associated with severity parameters in the ICU. This concerned mainly physical and emotional limitations, more than mental status, vitality, or pain. The same was observed in SARS-CoV-1 survivors, where lower SF-36 values were observed compared to healthy individuals and chronic disease patients at 6 months, similarly in the domains of physical and emotional health [17]. In addition to physical disability, ICU survivors commonly experience long-term mental health impairments. Post-traumatic stress disorder is present in 23% and persists for up to 5 years [20, 21]. Similar features were described in SARS-CoV-1 survivors [4]. Supplemental factors for altered emotional well-being included reduced access to family members during ICU stay, isolation, and fear of infecting others.

## Limitations of the study

The small size of the study is a limiting factor to generalize the present results, but it is partly counter-balanced by the homogeneity of the studied population.

To conclude, the majority of critical COVID-19 survivors are still disabled 3 months after ICU discharge and the severity of the sequelae are related to ICU disease severity score (APACHE II) and admission chest CT abnormalities. However, some patients have already completely recovered, providing hope that few of the critical COVID-19 survivors will experience long-term sequelae.

## Data Availability

The datasets used and/or analysed during the current study are available from the corresponding author on reasonable request.
